# Weighted spin torque nano-oscillator system for neuromorphic computing

**DOI:** 10.1038/s44172-023-00117-9

**Published:** 2023-09-20

**Authors:** T. Böhnert, Y. Rezaeiyan, M. S. Claro, L. Benetti, A. S. Jenkins, H. Farkhani, F. Moradi, R. Ferreira

**Affiliations:** 1 INL – International Iberian Nanotechnology Laboratory, Braga, Portugal; 2 Integrated Circuits and Electronics Laboratory, Department of Engineering, Aarhus University, Aarhus, Denmark

**Keywords:** Electronic and spintronic devices, Spintronics, Magnetic devices

## Abstract

Neuromorphic computing is a promising strategy to overcome fundamental limitations, such as enormous power consumption, by massive parallel data processing, similar to the brain. Here we demonstrate a proof-of-principle implementation of the weighted spin torque nano-oscillator (WSTNO) as a programmable building block for the next-generation neuromorphic computing systems (NCS). The WSTNO is a spintronic circuit composed of two spintronic devices made of magnetic tunnel junctions (MTJs): non-volatile magnetic memories acting as synapses and non-linear spin torque nano-oscillator (STNO) acting as a neuron. The non-linear output based on the weighted sum of the inputs is demonstrated using three MTJs. The STNO shows an output power above 3 µW and frequencies of 240 MHz. Both MTJ types are fabricated from a multifunctional MTJ stack in a single fabrication process, which reduces the footprint, is compatible with monolithic integration on top of CMOS technology and paves ways to fabricate more complex neuromorphic computing systems.

## Abstract (web summary)

Böhnert and colleagues demonstrate the weighted spin torque nano-oscillator, a spintronic circuit, as a basic programmable computing unit for neuromorphic computing systems. This circuit connects multiple spintronic devices with different functionalities in one circuit using a single fabrication process, which paves ways to fabricate more complex neuromorphic computing systems.

## Introduction

Von-Neumann based computing is predicted to reach an inevitable limit within 10 years due to fundamental limitations, such as enormous power consumption^1^. A promising strategy to overcome these barriers is massive parallel data processing, similar to the brain. These NCSs use many parallel processors (neurons) communicating using simple messages (spikes) or continuous interactions (oscillations) mediated by programmable memory units (synapses). The individual input signals are weighted in the synapses and mapped non-linearly into an output signal in the neuron. The close proximity of the processing and memory units and the non-volatile memory are characteristic for this architecture.

NCSs built in conventional CMOS improved in terms of energy efficiency and footprint, but are still far from the energy efficiency and footprint of the brain^2–4^. New hardware implementations of ultra-low energy and non-volatile artificial neurons could fill the performance gap, for example synapses and neurons have been implemented by memristors^5,6^, photonics^7,8^, spin Hall oscillators^9^, and MTJs^10–23^.

Spin-based computing is the most promising of the mentioned technologies with a small footprint and low power consumption due to the low intrinsic energy needed to manipulate nanomagnets^2,24–27^ and non-volatile information storage^28^. As demonstrated by the commercialization of MRAM technology, MTJ integrated on CMOS have shown to be scalable and robust^25^. The challenge for application in neuromorphic computing is to integrate the required functionality in a single scalable, energy- and space-efficient system. NCSs consist of multiple layers with each incorporating large numbers of neurons, which are all interconnected by many more synapses. Due to this complexity a reliable monolithic integration between spintronic components and CMOS is fundamental. Implementing synapses and neurons in a single CMOS-compatible process enables the monolithic integration on CMOS and improves the energy efficiency and density of the whole NCS due to a reduction of the footprint and the parasitic losses due to spatial separation.

Different types of devices can be used to implement synapses and neurons^29,30^. More specifically, neurons have been implemented using a wide range of MTJ micropillars and nanopillars: Using MTJs nanopillars with footprints as low as 0.008 µm^2^ as rectifiers requires modest RF inputs of 32 µW and results in small DC outputs of 0.2 nW^22^. By biasing the MTJ nanopillar with an additional DC current, the total input RF and DC input power value can be reduced below 10 µW^23^. Linearly magnetized spin-torque nano-oscillator (STNO) have an equally small footprint, but require 138 µW in input power and generate RF output powers of 5 nW^11^. While STNOs with a vortex magnetization state have larger output powers of a few microwatts and require input powers of a few milliwatts^20,31^. The synchronization of the multiple vortex STNOs can be used as activation of a neuron^10,21^. Thus, depending on the frequency and power requirements different solutions are preferable.

Here, we propose a spintronic circuit which we call weighted spin torque nano-oscillator (WSTNO) that consists of a resistive network interconnecting multiple MTJ devices with different functional characteristics determined by the MTJ geometry and made out of a single multifunctional MTJ stack. It combines non-volatile magnetic memories (MRAMs) acting as artificial synapses, and non-linear STNO with distinct threshold behavior acting as neurons. The resulting WSTNO is a building block with the required functional features required to implement a NCSs. A distinct feature of the WSTNO presented here compared to previously reported NCSs based on MTJs^15,17,19,20^ is the implementation of the non-volatility of the weights.

In literature one multifunctional MTJ stack (memory, oscillators, magnetic field sensor) is reported, which is optimized for memory applications (out-of-plane magnetization, thin free layer, low switching energy)^32^. In contrast, we prioritized the oscillator performance over the memory performance, in order to optimize the performance of the inference phase of neuromorphic applications. In this phase, the weights are written once for each neuromorphic application and the writing is not a fundamental part of the energy efficiency of the system computed over its life-time. We start from a vortex STNO stack^31,33,34^ (in-plane magnetization, thick free layer), due to the high signal-to-noise ratio, good reproducibility, low magnetic field requirements, large output power and low device to device variation compared to the alternatives, such as linear STNOs^11,35,36^ or spin Hall nano-oscillators^37,38^. We emphasize that optimized vortex STNOs are capable of exhibiting RF output powers in the range of 1-10 µW (−30 to −20 dBm) which is above the 1 µW threshold often cited as a minimum for integration of such devices with CMOS circuitry without major amplification requirements^39^. The magnetic behavior of the free layer is determined by the lateral dimensions of the nanopillars^40,41^. In nanopillars with a diameter above 300 nm the magnetization of the free layer forms a magnetic vortex state, which can be dynamically excited via spin transfer torque (STT) associated with the tunneling current generating an RF electrical signal of 200 to 400 MHz. Elliptical nanopillars with dimensions around 125 nm will have a uniformly in-plane magnetized free layer with additional shape anisotropy contribution that reinforces a bi-stable magnetization configuration appropriate for memory applications. Although the free layer thickness is optimized for vortex oscillators excited by spin transfer torques, the memory device can be easily switched by local magnetic fields generated by electric current lines.

The material stack of the devices used in this work is 5 Ta / 50 CuN / 5 Ta / 50 CuN / 5 Ta / 5 Ru / 6 Ir_0.2_Mn_0.8_ / 2.0 CoFe_0.3_ / 0.7 Ru / 2.6 Co_0.4_Fe_0.4_B_0.2_ / MgO wedge / 2.0 Co_0.4_Fe_0.4_B_0.2_ / 0.2 Ta / 7 NiFe / 10 Ta / 7 Ru (thicknesses in nm). The deposited MgO thickness was varied in a wedge across the wafer resulting in resistance area product (RxA) values between 3 and 20 Ωµm^2^ along the wafer. The results reported in this paper concern devices with RxA around 9 Ωµm^2^, which is typically the sweet spot in terms of maximizing oscillation power^35^. The nanodevices above this MgO thickness typically show tunnel magnetoresistance (TMR) values between 70 and 200%^35,42^. The MTJs presented in this work had a TMR of 60–70%.

For a matter of clarity, we demonstrate the concept of the WSTNO in a minimalistic network consisting only of two non-volatile MRAMs and one STNO. Each input is an analog voltage source that is weighted by the resistance state of the memory, which is either in the anti-parallel (AP) high resistance state or the parallel (P) low resistance state. The resulting input currents jointly excite the STNO into oscillation. In such a minimalistic system, only four possible configurations can be encoded in artificial synapses. A scaled up version of this minimalistic WTSNO with 10 MTJs in each synaptic input channel is presented in the Supplementary Note 1.

In conclusion, we present the concept and proof-of-principle of a WSTNO system, which combines different spintronic devices to create a basic programmable computing unit. On this basis, the experimental implementation of an artificial neuron is presented as a network of two MRAMs (synapses) and one STNO (neuron) nanofabricated from the same MTJ stack and only varying in lateral dimension. This allows the fabrication of dense MTJ nanopillar networks and is an advantage compared to artificial neurons consisting of different technologies for neurons and synapses. While the homogeneous integration of devices exploring different physical mechanisms and classes of materials is possible, in general, it requires compromises that are dictated by the compatibility of different materials and process flows that impact the density of integration. That is not the case here. The STNO output power was above 3 µW and frequency around 245 MHz in nanopillars of 300 nm diameter. Due to this high output power and low oscillation frequency, monolithic integration with CMOS technology is less challenging. The required footprint is about 0.017 µm^2^ per MRAM and 0.09 µm^2^ per STNO, which is very small compared to CMOS based artificial neurons that can have a footprint of 8 µm², but are usually far above this value^4,43^. Based on this proof-of-principle of a WSTNO system our long-term vision is to develop a unique platform for next-generation complex NCSs with improved performance compared to existing CMOS computing systems, filling the gap between the capability of current computers and the brain.

## Results

### The STNO characterization

**Fig. 1:**
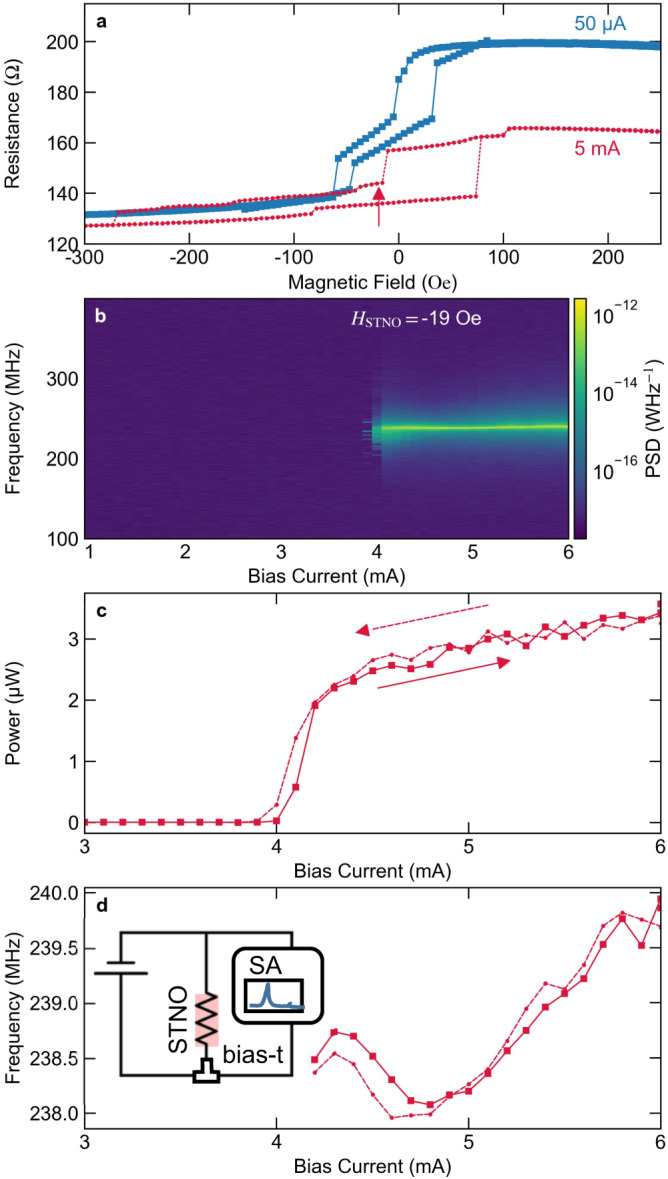
Spin-torque nano-oscillators (STNO) characterization overview. **a** Resistance of the STNO $({R}_{{{{{{\rm{STNO}}}}}}})$ as a function of an in-plane magnetic field at two bias currents. The arrow indicates the magnetic field conditions and the magnetic vortex state in which the auto oscillations are observed. **b** Power spectral density (PSD) of a 300 nm vortex STNO at an in-plane magnetic field of −19 Oe. **c**, **d** Integrated oscillation power $({P}_{{{{{{\rm{STNO}}}}}}})$ and resonance frequency obtained from a Lorenz fit of the PSD as a function of the bias current. The sweep was repeated in both directions as indicated by the arrows and shows no hysteretic behavior with the bias current. The insert in d shows the electric circuit of the measurement setup including the spectrum analyzer (SA).

The MTJ stack that is used in this work is optimized for consistent oscillations with output powers above 3 µW in an in-plane magnetic field of just ±20 Oe. This type of STNOs show a characteristic vortex magnetization state in the free layer. A DC bias current ${I}_{{{{{{\rm{STNO}}}}}}}$ is spin-polarized in the pinned layer and injected into the free layer of the STNO where it interacts through the STT effect, but also through the generated Oersted field and temperature. Above a critical current threshold, the magnetic vortex is excited into auto oscillation were the vortex core performs a gyrotropic motion. The gyration of the vortex core is sustained under the condition that the spin-transfer force is counterbalancing the damping. The micromagnetic behavior of vortex oscillators is well understood qualitatively as described elsewhere^31,33,34^. This circular orbit in reference to the in-plane magnetized reference layer creates a resistance oscillation due to the TMR effect of the MTJ. Thus, the magnetic state can be probed by measuring the electrical resistance as a function of the external magnetic field, as shown in Fig. 1a. The resulting AC oscillation power is measured using a spectrum analyzer, as shown in Fig. 1b^31^. A Lorenz fit of the observed oscillation peak is used to determine the integrated power (${P}_{{{{{{\rm{STNO}}}}}}}$) and the oscillation frequency shown in Fig. 1c, d. The ${P}_{{{{{{\rm{STNO}}}}}}}$ shows a distinct non-linear threshold behavior as a function of ${I}_{{{{{{\rm{STNO}}}}}}}$. The threshold is called critical current and is the point at which the damping is compensated by the STT effect^44^. However, the influence of grains can result in deviation from the theory^31,33,45^. The STNOs used in this work have a critical current of around 4 mA with an almost linear ${P}_{{{{{{\rm{STNO}}}}}}}$ increase above that value up to 3 µW for ${I}_{{{{{{\rm{STNO}}}}}}}=6{{{{{\rm{mA}}}}}}$ and an oscillation frequency around 240 MHz. Typically, the oscillation frequency is increasing as a function of the bias current until it approaches a maximum in these type of oscillators^33^. This particular STNO shows an almost constant frequency response, but most likely because the oscillation only occurred close to the maximum frequency were the frequency increase is small. The typical behavior measured on a set of seven STNOs of 300 nm diameter at a bias of 5 mA showed a frequency of 245 ± 11 MHz, a power of 1.68 ± 0.80 µW and a linewidth of 0.7 ± 0.5 MHz. These variations are not are not excessively high and should not affect the overall system performance.

The maximum ${P}_{{{{{{\rm{STNO}}}}}}}$ is about 4 µW before the breakdown of the MgO layer. During the measurement, an external magnetic field of −19 Oe is applied in an in-plane direction with no out-of-plane components in order to obtain oscillations, which is excellent for application as this field strength can be easily obtained by local magnetic fields.

### MRAM characterization

**Fig. 2:**
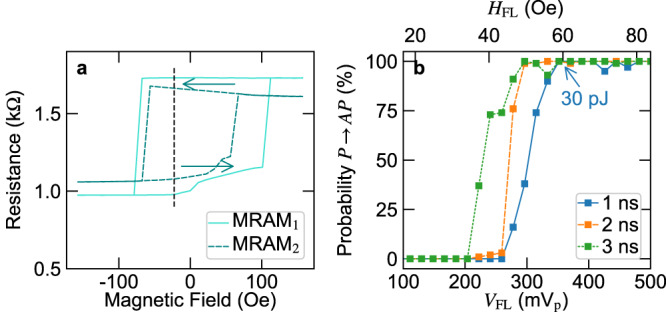
Magnetic memory (MRAM) characterization overview. **a** Resistance versus magnetic field transfer curves of elliptical MRAMs (75 nm x 225 nm). The magnetic field (*H*) was kept at −22 Oe to compensate for the magnetic field offset during the weighted spin torque nano-oscillators characterization. These curves were obtained at a bias current of 10 µA. **b** Switching probability overview of an elliptical MRAM (100 nm x 200 nm) from parallel to anti-parallel state as a function of the pulse voltage and magnetic field amplitude applied to the field line (*V*_FL_ and *H*_FL_). Each point is the result of 100 switching attempts with a single pulse of a few nanosecond length. A single 30 pJ pulse of 1 ns length is able to switch the MRAM from parallel (P) to anti-parallel (AP) state with a probability of 100%.

The challenge in fabricating the MRAMs is to obtain a bi-stable resistance state in an MTJ stack that was optimized to have a vortex ground state. This is achieved by introducing shape anisotropy through an elliptical shaped footprint. The MRAMs have a mostly square-like hysteresis loop with defined P and AP resistance states at zero field and two different coercive fields, as shown in Fig. 2a. In the presented samples, this coercive field difference is due to device-to-device variations, but it can be designed by the aspect ratio variation of the elliptical shaped footprint. The difference in the coercive field allows programming the MRAMs into all of the various resistance state combinations (P-P, P-AP, AP-P, and AP-AP) using minor loops of an externally applied magnetic field. To increase the energy efficiency, the individual MRAMs can be programmed through short pulses in independent magnetic field lines 1.0 µm above the devices, which generates a magnetic field of about 0.67 ${{{{{\rm{kOe}}}}}}{{{{{{\rm{A}}}}}}}^{-1}$ (in field lines with a 3 µm width). To study the required energy to program the 100 nm x 200 nm MRAM nanopillar we applied one hundred 1 ns, 2 ns, and 3 ns switching pulse and evaluated the switching probability for each pulse length as a function of the pulse amplitude. A switching probability of 100% is obtained for 1 ns pulses above an amplitude of 350 mV, as shown in Fig. 2b, which corresponds to an energy of 30 pJ. This value is within the typical range of energies cited for comparable field switching MRAM cells, and reasonable considering that this MTJ stack was not optimized for switching efficiency^46,47^. The average P and AP resistance of 56 elliptical MRAM devices (75 nm × 225 nm) across the 200 mm wafer was 740 ± 277 Ω and 1445 ± 356 Ω, respectively. These variations can be reduced with optimization of the fabrication process as demonstrated for example for STT-MRAM devices^48^.

### WSTNO characterization

**Fig. 3:**
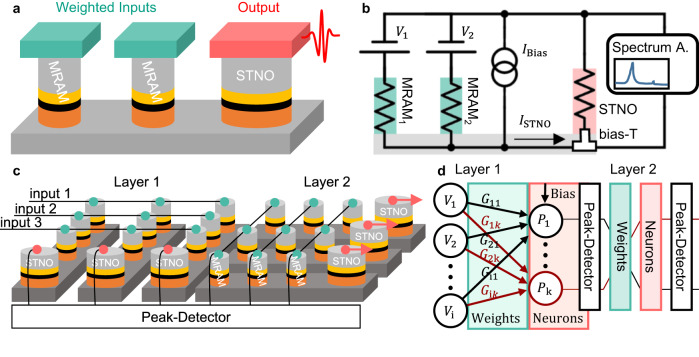
Weighted spin torque nano-oscillators (WSTNO) schematics. **a** WSTNO consisting of two magnetic memories (MRAM) as weights and a larger nanopillar as a spin torque nano-oscillator (STNO). The MRAMs are the non-volatile weights and the STNO is responsible for the non-linear transfer function of the neuron. **b** Schematic of the DC circuit used in the demonstration presented here. The input voltages (*V*_1_ and *V*_2_) are weighted by the MRAMs together with the bias current (*I*_Bias_) and excite the STNO. The STNO excitation current (*I*_STNO_) is converted non-linearly into an oscillating output power. **c** Example schematic showing the layout of two layers with each 3 inputs and 3 outputs demonstrating the scaling of the suggested neuromorphic circuit consisting of a crossbar array of MRAMs and STNO elements at the output of each layer. The number of inputs, outputs and layers can be scaled and each should increase the footprint linearly. The peak detector shown here is the interface circuit between layers and could be implemented in CMOS as discussed in the manuscript. **d** Network representation of the suggested neuromorphic circuit. Shown are the input voltages (*V*_i_), the conductance as the weights (*G*_ik_) of the synapses (MRAMs), the neuron (STNO) with output power (*P*_k_), the peak detector and the simplified subsequent layer.

The proof-of-principle implementation of the WSTNO with two MRAMs and one STNO, as shown in Fig. 3a. For the WSTNO characterization, the input signals ${V}_{1}$ and ${V}_{2}$ are applied to the two MRAMs and a current source (${I}_{{{{{{\rm{Bias}}}}}}}$) is added in parallel, as shown in the electric circuit in Fig. 3b. The total STNO excitation current $({I}_{{{{{{\rm{STNO}}}}}}})$ is given by (1). The current flowing through each MRAM $({I}_{i})$ results from the input voltage (${V}_{i}$) and the conductance of each MRAM (${G}_{i}={R}_{i}^{-1}$), but is slightly reduced by a leak current as expressed in (2). The current flow is determined by the relation of STNO resistance $({R}_{{{{{{\rm{STNO}}}}}}})$ to the MRAM resistances $({R}_{{{{{{\rm{MRAM}}}}}}})$. The leakage is minimized in the limit of ${R}_{{{{{{\rm{STNO}}}}}}}\ll {R}_{{{{{{\rm{MRAM}}}}}}}$, which results in the typical artificial neuron output Eq. (3)^49^. The MRAMs weight and accumulate the inputs, which are then non-linearly converted into the output power ${P}_{{{{{{\rm{STNO}}}}}}}$.

1 $${I}_{{{{{{\rm{STNO}}}}}}}=\left(\sum {V}_{i}{G}_{i}+{I}_{{{{{{\rm{Bias}}}}}}}\right){R}_{{{{{{\rm{parallel}}}}}}}{R}_{{{{{{\rm{STNO}}}}}}}^{-1}$$

2 $${I}_{i}={V}_{i}{G}_{i}-{I}_{{{{{{\rm{STNO}}}}}}}{R}_{{{{{{\rm{STNO}}}}}}}{G}_{i}$$

3 $$y=f\left(\sum {V}_{i}{G}_{i}+{I}_{{{{{{\rm{Bias}}}}}}}\right),$$

with the resistance of all MTJs in parallel given by ${R}_{{{{{{\rm{parallel}}}}}}}={\left({\sum }_{i}{R}_{i}^{-1}+{R}_{{{{{{\rm{STNO}}}}}}}^{-1}\right)}^{-1}$ and $f$ being the non-linear transfer function, which is here the ${P}_{{{{{{\rm{STNO}}}}}}}$ behavior. ${P}_{{{{{{\rm{STNO}}}}}}}$ is a function of ${I}_{{{{{{\rm{STNO}}}}}}}$ and resembles the shape of the commonly used leaky ReLU transfer function (see Fig. 1c) with additional step at the critical current^50^.

**Fig. 4:**
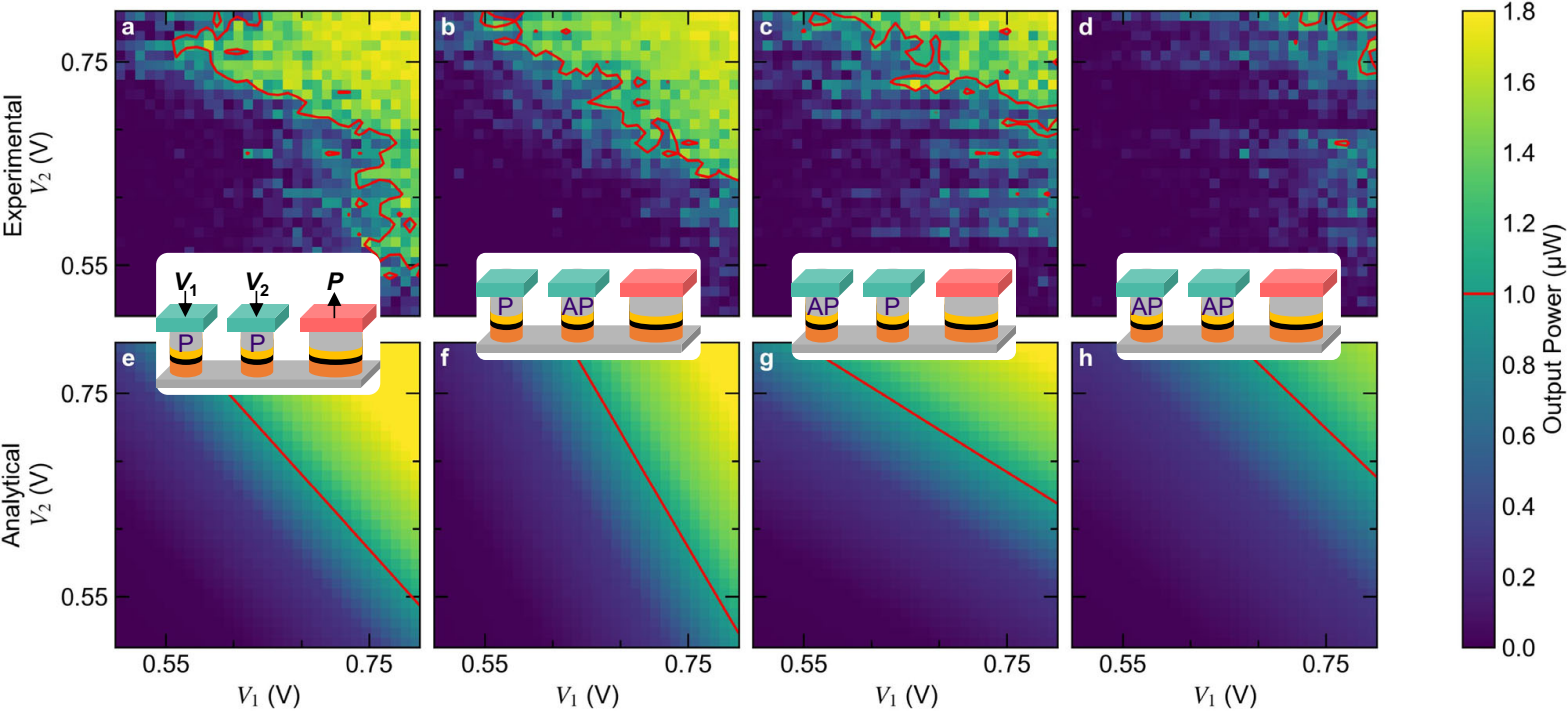
Weighted spin torque nano-oscillator (WSTNO) characterization overview of the input state combinations. **a–d** Measured oscillation power (*P*) of the WSTNO as a function of the input voltages (*V*_1_ and *V*_2_) for each possible combination of the non-volatile states of the MRAMs: parallel (P) and anti-parallel (AP). Each figure represents a computation with two analog inputs and one analog output according to (3). The contour of 1 µW output power is shown in red. **e–h** Analytical prediction of the WSTNO behavior as a function of the two input voltages for each state combination. This prediction is obtained using formula (3) and the characterizations of the individual MRAMs and the STNO.

In Fig. 4, we present the integrated ${P}_{{{{{{\rm{STNO}}}}}}}$ of one WSTNO as a function of the two input voltages applied to two MRAMs. The ${P}_{{{{{{\rm{STNO}}}}}}}$ shows a non-linear behavior weighted by the non-volatile input states (P or AP) of each MRAM, shown in the subfigures Fig. 4a–d. The non-linear behavior of ${P}_{{{{{{\rm{STNO}}}}}}}$ is clearly shown for each of the possible input states. The behavior of the artificial neuron is analytically predicted, as shown in Fig. 4e–h, based on Eq. (3) and the experimental characterizations of the individual MRAMs and the STNO, specifically the resistance values of all components and the behavior of ${P}_{{{{{{\rm{STNO}}}}}}}$ as a function of ${I}_{{{{{{\rm{STNO}}}}}}}$. The WSTNO is programmed by minor loops of the applied magnetic field, which sets each MRAM in the respective state. For optimal oscillation conditions during the WSTNO characterization an in-plane magnetic field of −19 Oe and ${I}_{{{{{{\rm{Bias}}}}}}}$ of 3.9 mA are applied to the STNO. A comparable magnetic field of −22 Oe was applied to the MRAM during operation. Thus, a magnetic field of similar magnitude is applied to all spintronic devices during the WSTNO characterization.

## Discussion

The measured ${P}_{{{{{{\rm{STNO}}}}}}}$ of the WSTNO follows the predicted behavior reasonably well. There are some deviations in the mixed resistance states (P-AP, AP-P) and a structure along the scan direction (parallel to the y-axis) of the experiment is observed. We believe this is due to heating effects and small irreversible resistance changes of the nanometer-sized MRAMs during the four-hour measurement required to gather the data in Fig. 4 with high resolution. We believe the stability could be improved by reducing the RxA or increasing the lateral size of the MRAM devices. Nevertheless, the results clearly show the ability to program and perform computations through this WSTNO-based implementation of an artificial neuron.

As an example, we can classify the artificial neuron as active above a characteristic threshold power of ${P}_{{{{{{\rm{STNO}}}}}}} >$ 1 µW as marked by the contour in Fig. 4. In the P-P configuration the WSTNO activates above a combined voltage of both inputs (*V*_1_ + *V*_2_) of 1.35 V, as shown in Fig. 4a. However, programmed to the AP-AP configuration, the WSTNO activates at a combined input (*V*_1_ + *V*_2_) above 1.46 V, as shown in Fig. 4d. In the mixed states (P-AP, AP-P) one of the inputs will be weighted less and the STNO will show an asymmetric activation behavior, as shown in Fig. 4b, c, f, g. The specific activation range can be shifted using the ${I}_{{{{{{\rm{Bias}}}}}}}$ value.

The non-volatile MRAMs can be programmed by 1 ns current pulses of 30 pJ. Although not directly comparable, this performance looks promising considering that state of the art networks such as Loihi by Intel has a system level consumption of 120 pJ energy per synaptic update, which required 6.1 ns^51^. Due to the non-volatility the static power consumption of WSTNO is almost zero, while we estimate the required oscillation energy of a single STNO neuron with about 25 pJ in 5 ns operating time (see Table 1 and Supplementary Note 2). Although not directly comparable, this looks promising considering the reported energy consumption of a single neuron in Loihi is 81 pj/52 pJ when it is active/inactive. Compared to vortex STNOs based neuron implementation in literature the footprint, frequencies and input powers are in a comparable range, while the output power is about an order of magnitude larger in the presented devices^21^. From a fundamental point of view, there is wide room for improvement in terms of footprint and input power particularly by reducing the STNO diameter as suggest in literature^20^.

**Table 1:** Overview of the characteristic of the devices presented in this work.

Device	Area (µm^2^)	Switching/Operation time (ns)	Power consumption (pJ)
MRAM/Synapse	0.015	1	30
STNO/Neuron	0.07	5	25
WSTNO/2 Synapses + Neuron	0.037	5	27.5

The area is the footprint area of each nanopillar and the combined area for the weighted spin torque nano-oscillator (WSTNO). The switching time describes the programming operation of the magnetic memory (MRAM) and the operation time describes the computation of the spin torque nano-oscillator (STNO) and the WSTNO. Further details are described in Supplementary Note 2.

Device-to-device variations, as mentioned in the MRAM and STNO characterization, can have an noticeable effect in large networks and the fabrication processes should be further optimized with this in mind. We believe these variations can be partially compensated during the training phase and by the CMOS circuit. For example, lowering the weights or adjusting the bias current can correct a low critical current value. Certainly, the remaining variations might affect the quantized weight resolution of the WSTNO and the final output value.

As an outlook, a future WSTNO system will have multiple distinct weighting levels (resistance states)^52^, many more inputs (number of input voltages), and multiple detection levels (output power of the STNO). Multiple of these basic elements can compute in parallel in layers and multiple layers follow each other in series (see Fig. 3a–d) to perform more complex operations in a neural network^13^. To increase the distinct weighting levels, additional MRAMs of varying sizes can be added in series to extend the weighting states to higher binary numbers. First results of this approach are shown in Supplementary Fig. 2. The monolithic integration of WSTNOs on CMOS will be an important step to reach this level of complexity.

The presented WSTNO system receives DC voltages as inputs and converts them to a current depending on the state of the MRAM element. A DC bias current is added, as shown in Fig. 3. The combined current excites the STNO non-linearly into oscillation. This bias current is set below the critical current of the oscillation of a WSTNO. Hence, no current through the MRAMs means no oscillation and negligible output power. By applying the inputs to the MRAMs, for ${I}_{{{{{{\rm{STNO}}}}}}}$ (see Eq. (1)) higher than a threshold current, the corresponding WSTNOs will oscillate which means the corresponding neuron is firing. Then the AC voltage across STNO will be sensed using a peak detector (PD) circuit and will be converted to a binary output. In case there is no oscillation for an STNO, the output of PD will be ‘0’ (no spike), otherwise, it will be ‘1’ (representing a spike). The PD can be a CMOS inverter-based low noise amplifier with a sub-µW power consumption^53^, which is followed by a comparator with a power consumption lower than 200 µW^54^. As a result, the whole proposed neural network works similar to a spiking neural network (SNN). To demonstrate the feasibility on a network level we simulated a simple binary network of 10 STNOs with 20 weights each in Cadence Virtuoso with the task of recognizing digits represented by 4 × 5 pixels pictures. The successful results of this simulations are shown in Supplementary Note 3.

Alternatively, an artificial neural network that computes analog output signals can be implemented using an envelope detector instead of the PD at the cost of speed and power. Similarly, the spin diode effect or wide-band diode effects in vortex STNOs can be used as frequency dependent rectifiers^55–57^ considering sufficient amplification by the CMOS circuit. This conversion stage offers an opportunity to tackle the scaling problem by interconnecting subsequent layers through a single wave-guide that interconnects the multiplexed frequency signal of all outputs to all inputs^15,20^. Another opportunity for optimization is local optical heating for example by integrating vertical-cavity surface-emitting laser (VCSEL) structures for the temporary reduction of the critical currents^12,58^.

## Methods

### MTJ stack

The MTJ stack containing two CoFeB layers sandwiching an MgO tunneling barrier was deposited on Si/200 nm SiO_2_ substrate in a physical vapor deposition system with a base pressure of 10^–9^ Torr. The top CoFeB is in close proximity to a NiFe layer that creates a magnetic vortex structure. This bilayer acts as the free layer of the MTJ. The bottom CoFeB layer is called pinned layer and is coupled to the synthetic antiferromagnet that forces the magnetization to point uniformly towards the reference direction. If the free layer is aligned with the reference direction the MTJ is in the parallel configuration with low electrical resistance, while in the other extreme it is in the anti-parallel configuration with high resistance. More details on similar devices are found elsewhere^59,60^.

### MTJ nanopillar fabrication

The nanopillar fabrication can be separated in four parts: nanopillar patterning, bottom electrode definition, planarization, and top electrode definition. The electron beam lithography was used to define the nanopillar, otherwise direct write laser lithography was used. The dielectric material that surrounds the nanopillars was SiO_2_. The top contact was a 450 nm layer of AlSiCu alloy and the field line was an 800 nm layer of AlSiCu alloy. Ion beam etching was used for all the etching purposes. We fabricated and studied not only individual devices of various shapes, sizes and MgO layer thicknesses, but also complete WSTNO systems consisting of two MRAMs and one STNO each. Further details of similar fabrication processes are described elsewhere^35,38,42^.

### WSTNO setup

For the presented WSTNO experiments we used individual 300 nm diameter circular nanopillars as STNOs and two 75 nm × 225 nm elliptical nanopillars as MRAMs. Two interconnected probe stations with multiple micro positioners, three Keithley 2400 source measure units as inputs, a bias-T, and an Agilent E4446a spectrum analyzer to integrate the output power were used. The spectrum analyzer was used with a resolution bandwidth of 39 kHz. In each probestation a separately controlled external in-plane magnetic field was applied. The use of two probestation and individual devices gave us full control of the experimental parameters and allowed us to freely combine different geometries. No further amplification was necessary during the experiments. The oscillation power and frequency values were obtained by a Lorentz fit of the measured spectrum. The stated oscillation power values are as delivered to the 50 Ω load without any mismatch considerations.

### Switching probability setup

A 100 nm x 200 nm nanopillar with a so-called field line a few 100 nm above the device was used for the analysis of the switching probability. We used a Keysight M8190A arbitrary waveform generator and a 42 dB amplifier to inject a single pulse into the 4 Ω field line. The pulse shape was monitored on a high input impedance oscilloscope. Pulses down to 1 ns pulse with an around ~0.5 ns rise time were obtained in this setup. The switching experiment was repeated 100 times at each condition to estimate the switching probability. The given pulse amplitudes and powers are measured at the field line, thus, losses due to mismatching of the electric circuit are not included.

### Supplementary information

Supplementary Information

## Data Availability

The data that support the findings of this study are available from the corresponding author upon reasonable request.
