# Supplementary material for: Weighted spin torque nano-oscillator system for neuromorphic computing
Source: Commun Eng. 2023 Sep 20;2:65. doi: 10.1038/s44172-023-00117-9 (PMC10955825; doi:10.1038/s44172-023-00117-9)
Supplement: Supplementary file 1 — Supplementary Information [file 44172_2023_117_MOESM1_ESM.pdf]

Weighted Spin Torque Nano-Oscillator System for Neuromorphic Computing

T. Böhnert[1*], Y. Rezaeiyan[2], M. Claro[1], L. Benetti[1], A. S. Jenkins[1], H. Farkhani[2], F. Moradi[2], R. Ferreira[1]

[1] INL – International Iberian Nanotechnology Laboratory, Av. Mestre José Veiga s/n, Braga 4715-330, Portugal
[2] Integrated Circuits and Electronics Laboratory, Aarhus University, Aarhus, Denmark
Correspondence and requests for materials should be addressed to T.B. (email: tim.boehnert@inl.int)

Supplementary Information

Supplementary Note 1: Multistate weights

In order to move from binary weights with two states to higher number of states we increase the number of MRAM elements in series. The WSTNO structure for two states is shown in Supplementary Figure 1a and a WSTNO structure with 12 states (11 MRAMs) is shown in Supplementary Figure 1b. In this structure all MRAM elements are of the same size, but with varying switching fields. Thus, each switching will lead to a resistance jump of around 100 Ω, as shown in Supplementary Figure 2, leading to 12 resistance states independent of the order of switching. This scheme is often described as thermometer scale. Alternatively, each MRAM elements can have twice the size of the previous element and be individually switched. This leads to a binary scale with 2^N states, where N is the number of MRAM elements. In this scheme the order of switching has to be controlled on an individual device basis. Further optimization is ongoing in order to improve the control and reliability of these multistate devices.

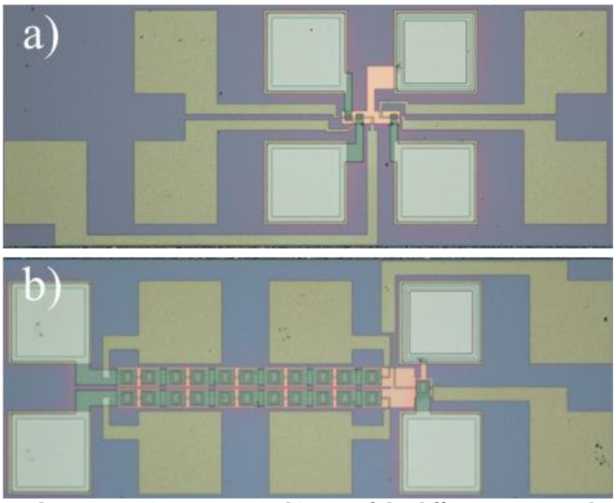

Supplementary Figure 1. Optical image of the different WSTNO device structures. a Device structure of the WSTNO including bottom contact, top contact, two MRAMs, STNO and field lines. **b** Device structure of the multistate WSTNO including bottom contact, top contact, 22 MRAMs, STNO and field lines.

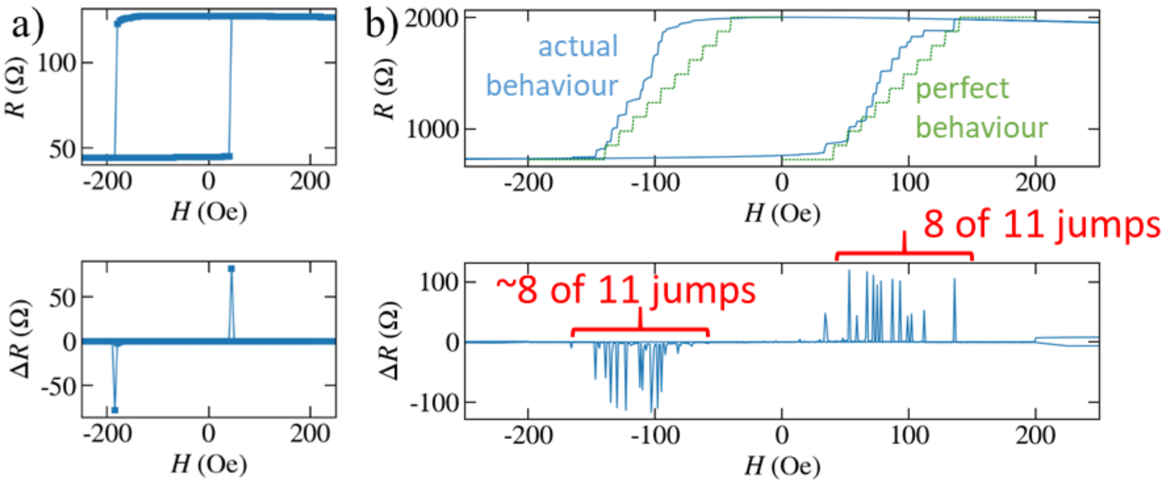

Supplementary Figure 2. Electric measurement of the switching behavior of the weights in different configurations. a Resistance (*R*) and height of the resistance jumps of a single MRAM element as a function of the magnetic field (*H*). Two well defined resistance jumps are visible. **b** Resistance and height of the resistance jumps of the multistate weights (multiple MRAM elements) as a function of the magnetic field. Multiple resistance jumps are detected. In comparison to the perfect behavior some deviations are visible. The switching fields are not equidistant and the resistance jump amplitude for some devices is smaller than expected. However, the majority of the devices is behaving as expected.

Supplementary Note 2: Power consumption of the WSTNO system

To get an idea about the performance of the WSTNO concept we try to compare the proposed WSTNO with CMOS leadership neuromorphic chip, Loihi by Intel. When compared with CMOS technology, the power consumption can be divided into two categories:

Training power consumption: The training power consumption of WSTNO is comparable with its CMOS counterpart. It is difficult to compare the exact power consumption due to a lack of information about the power consumption of the peripheral circuits needed for switching MRAMs. For an estimation we can do the following comparison. Loihi by Intel[1] consumes 120 pJ, while this energy is 30 pJ per MRAM for WSTNO (of course the energy consumption of peripheral CMOS circuit for performing the write operation should be added to this value). In terms of speed, the time per synaptic update in Loihi is 6.1 ns while it can be as low as 1 ns for WSTNO.

Processing power consumption: The static power consumption of WSTNO is almost zero. It means after training the network there is no static power consumption in this device for keeping the stored weights of synapsis on MRAMs. However, its CMOS counterpart (either a digital CMOS design that uses SRAM or an analog CMOS design that utilizes a capacitor for storing the weight) consumes static power consumption. It means even after training, for keeping the data, continuous power should be consumed to compensate for the leakage through the parasitic capacitors. The reported energy consumption of a single neuron in Loihi is 81 pj/52 pJ when it is active/inactive. However, the neuron energy consumption depends on both the energy consumption of the STNO as a neuron and the energy consumption of the CMOS readout circuit to be used for sensing this oscillation. If we suppose that the CMOS readout circuit is able to detect the oscillation within a few nanoseconds, considering the 5 mA critical current of STNOs, the energy consumption of STNO can be estimated in the order of 200 Ω x $(5\text{ mA})^2$ x 5 ns = 25 pJ. Even by considering the energy consumption of the CMOS readout circuit, the total energy consumption of the WSTNO is comparable with Loihi. The time per neuron activation is around 8.4 ns for Loihi and it will take a similar time (a few ns) for the CMOS readout circuit to detect the WSTNO oscillation. We estimate the power consumption of a computation of the WSTNO with bias current of 4 mA applied only to the STNO and 0.5 mA applied to each MRAM element (1k Ω) and the STNO (200 Ω). As a rough estimation this results in a WSTNO consumption of roughly (200 Ω x $(5\text{ mA})^2$ + 1000 Ω x $(0.5\text{ mA})^2$ + 1000 Ω x $(0.5\text{ mA})^2$) x 5 ns = 27.5 pJ per computation.

Supplementary Note 3: Digital Digit Classification

The proposed WSTNO-based neural network is shown in Supplementary Figure 3a. In a trained neural network, the same inputs usually trigger the same chain reactions in such a way to fire the same neuron at the output layer. The proposed neural network includes only one layer to classify 10 digits. For this classification task, the input is 4x5 pixels digits pictures (Supplementary Figure 3b), which are digitized using a 1-bit voltage Digital to Analog Converter (DAC) fed into the network. Therefore, inputs of the network can be either '0' or '1' values. These values represent whether a pixel is gray ('0') or blue ('1'). This binary input is converted to a current value to contribute to the STNO biasing through MRAMs. The whole network includes 10 STNOs with 20 MRAMs as a weighted neuron. Each STNO corresponds to a digit (0-9) and the best weights are found using equation (1) and equation (3) of the main manuscript to guarantee that only one neuron (the corresponding STNO) oscillates or fires for each input pattern. Supplementary Figure 3c shows the simulation results obtained using Cadence Virtuoso. As it is seen, only the corresponding neuron fires for the corresponding input.

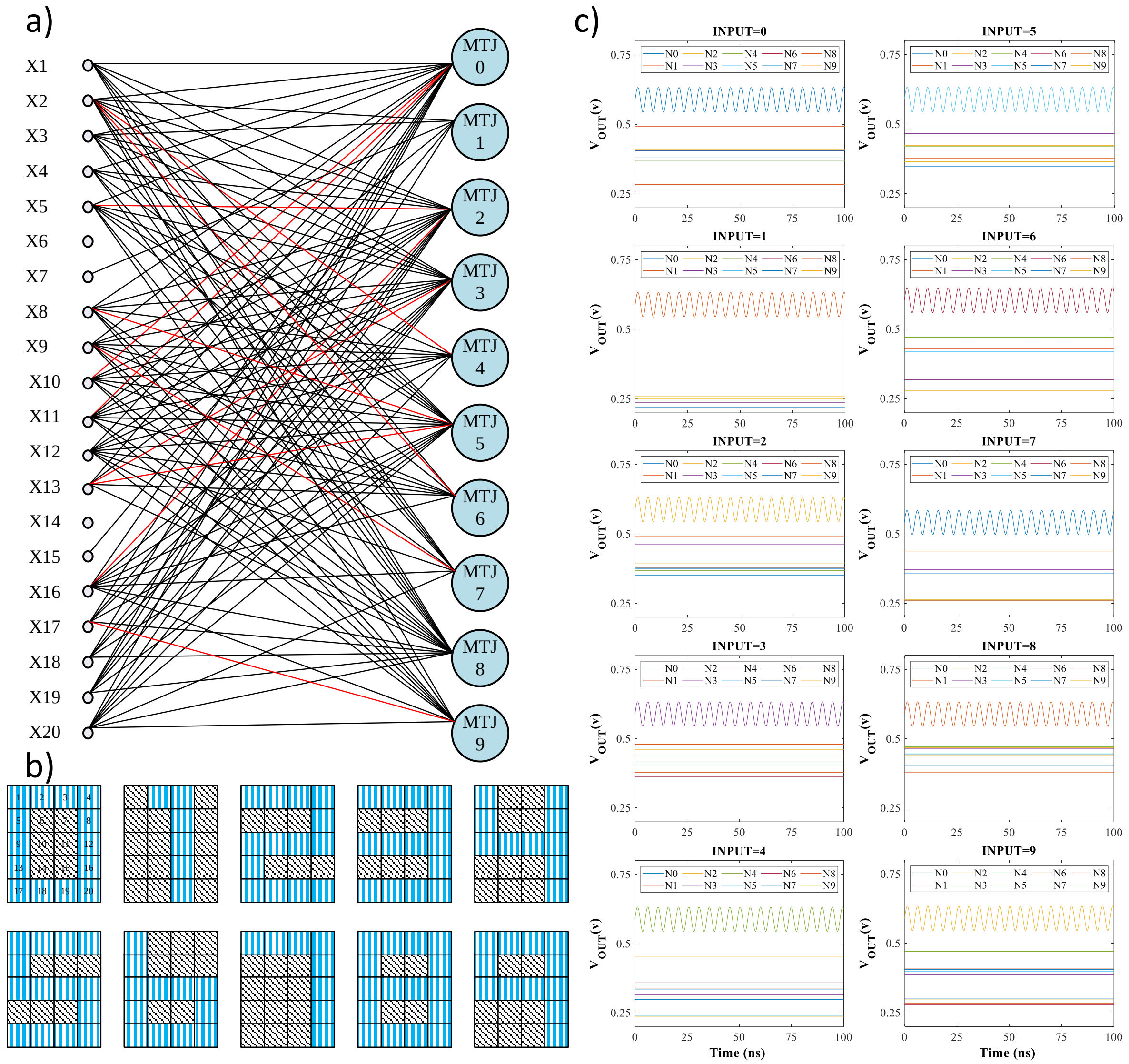

Supplementary Figure 3. Simulation of the proposed WSTNO-based neural network. a The neural network with 10 STNO neurons with 20 DC voltages as inputs, **b** 10 digit input patterns of the network, and **c** the simulation results of the proposed WSTNO-based neural network

Supplementary References

1. Davies, M. et al. Loihi: A Neuromorphic Manycore Processor with On-Chip Learning. *IEEE Micro* **38**, 82–99 (2018).
